# Confirmatory Factor Analysis of the WHO Violence Against Women Instrument in Pregnant Women: Results from the BRISA Prenatal Cohort

**DOI:** 10.1371/journal.pone.0115382

**Published:** 2014-12-22

**Authors:** Marizélia Rodrigues Costa Ribeiro, Maria Teresa Seabra Soares de Britto e. Alves, Rosângela Fernandes Lucena Batista, Cecília Cláudia Costa Ribeiro, Lilia Blima Schraiber, Marco Antônio Barbieri, Heloisa Bettiol, Antônio Augusto Moura da Silva

**Affiliations:** 1 Department of Medicine III, Federal University of Maranhao, Sao Luis, Brazil; 2 Department of Public Health, Federal University of Maranhao, Sao Luis, Brazil; 3 Department of Odontology, Federal University of Maranhao, Sao Luis, Brazil; 4 Department of Preventive Medicine, Faculty of Medicine, University of Sao Paulo, Sao Paulo, Brazil; 5 Department of Pediatrics, Faculty of Medicine of Ribeirao Preto, University of Sao Paulo, Ribeirao Preto, Brazil; Waseda University, Japan

## Abstract

**Background:**

Screening for violence during pregnancy is one of the strategies for the prevention of abuse against women. Since violence is difficult to measure, it is necessary to validate questionnaires that can provide a good measure of the phenomenon. The present study analyzed the psychometric properties of the World Health Organization Violence Against Women (WHO VAW) instrument for the measurement of violence against pregnant women.

**Methods:**

Data from the Brazilian Ribeirão Preto and São Luís birth cohort studies (BRISA) were used. The sample consisted of 1,446 pregnant women from São Luís and 1,378 from Ribeirão Preto, interviewed in 2010 and 2011. Thirteen variables were selected from a self-applied questionnaire. Confirmatory factor analysis was used to investigate whether violence is a uni-or-multidimensional construct consisting of psychological, physical and sexual dimensions. The mean-and-variance-adjusted weighted least squares estimator was used. Models were fitted separately for each city and a third model combining data from the two settings was also tested. Models suggested from modification indices were tested to determine whether changes in the WHO VAW model would produce a better fit.

**Results:**

The unidimensional model did not show good fit (Root mean square error of approximation [RMSEA]  = 0.060, *p<*0.001 for the combined model). The multidimensional WHO VAW model showed good fit (RMSEA = 0.036, *p* = 0.999 for the combined model) and standardized factor loadings higher than 0.70, except for the sexual dimension for SL (0.65). The models suggested by the modification indices with cross loadings measuring simultaneously physical and psychological violence showed a significantly better fit compared to the original WHO model (*p*<0.001 for the difference between the model chi-squares).

**Conclusions:**

Violence is a multidimensional second-order construct consisting of psychological, physical and sexual dimensions. The WHO VAW model and the modified models are suitable for measuring violence against pregnant women.

## Introduction

The expression “violence against women” involves complex, dynamic and historically determined phenomena. Abuse of women represents gender violence produced by unequal power relations, reflecting primacy of males over females [1]–[3].

Highly prevalent, violence against women has been pointed out as a phenomenon that is difficult to measure. There is variation on what types of acts are considered violent by a particular woman or a group of women. There is also various definitions and types of violence and/or methodological diversity across studies [4], [5].

The use of different terminologies to express the various forms of abuse, types of study, places and periods of women's life when they are interviewed, sample size, screening instruments, perpetrators and modes of questionnaire application, among other aspects, have impaired the comparability of the results of different investigations regarding violence against women [6]–[9].

In order to minimize the methodological problems and to permit transcultural comparisons, the World Health Organization carried out the study Violence Against Women (WHO VAW) [2]. Thirteen questions were elaborated to investigate the psychological, physical and sexual types of violence and were included in the questionnaire WHO Multi-country Study on Women's Health and Life Events. More than 24,000 women aged 15 to 49 years were interviewed at 15 locations in 10 countries between 2000 and 2003, corresponding to random samples representative of the populations [10].

The WHO VAW instrument, composed of 13 questions measuring psychological (four), physical (six) and sexual (three) violence, showed good internal consistency, indicating that it provides a reliable and valid measure of these types of violence [10].

In Brazil, the WHO VAW questionnaire was validated using data from the city of São Paulo (1,172 women) and from 15 municipalities located in the Wooded Zone of Pernambuco (1,473 women). Exploratory factor analysis showed that this instrument is suitable for the estimation of gender violence perpetrated by an intimate partner, with high internal consistency and capacity to discriminate between emotional, physical and sexual violence within different social contexts [11].

Another study also used exploratory factor analysis to validate the WHO VAW questionnaire in a random sample of 573 Swedish women interviewed at the age of 18 to 65 years, and concluded that this screening instrument has good construct validity and internal consistency. The investigators pointed out the lack of studies at the international level with similar objectives, and only cited the Brazilian study of WHO VAW validation [12].

However, exploratory factor analysis analyzes the pattern of correlations between the variables investigated and uses these patterns to group them into factors. Model fit is not evaluated and it is not possible to test the hypothesis that a certain set of relationships between the observed variables and the proposed underlying construct exists. In turn, confirmatory factor analysis is a technique of multivariate statistical analysis that permits the investigator to analyze the pattern of correlations between the observed variables (or indicators) and to test hypotheses, in addition to proposing alternative models to the initial one [13], [14].

Confirmatory factor analysis for the validation of the WHO VAW instrument has not been used yet and no studies were detected validating this instrument during the gestational period with the use of the self-applied questionnaire. Thus, the objective of the present study was to analyze the psychometric properties of the WHO VAW instrument in a sample of pregnant women in order to determine whether violence is a uni-or-multidimensional construct consisting of psychological, physical and sexual dimensions, using confirmatory factor analysis.

## Methods

The present investigation is part of the Brazilian Ribeirão Preto and São Luís Birth Cohort Studies (BRISA in the Portuguese acronym), carried out by the Federal University of Maranhão (UFMA) and by the Faculty of Medicine of Ribeirão Preto, University of São Paulo (FMRP/USP) in two municipalities with contrasting socioeconomic indicators: São Luís (State of Maranhão) and Ribeirão Preto (State of São Paulo). The study is part of a large research project, aimed at evaluating risk factors for preterm birth in a convenience sample of pregnant women selected during the first 20 weeks of pregnancy.

### The municipalities of Ribeirão Preto and São Luís

The municipality of Ribeirão Preto is located in the state of São Paulo, in the Southeast region of Brazil. In 2010, its population was 604,682 inhabitants, with a 99.72% urbanization rate and a mean per capita family income of R$ 1,314.04 (approximately 728 American dollars) [15]. In this city, in 2011, 77.3% of all pregnant women attended at least seven prenatal care visits [16].

The municipality of São Luís, capital city of the state of Maranhão, is located in the Northeast region. In 2010, its population was 1,014,837 inhabitants, with a 94.5% urban population rate and a mean per capita family income of R$ 805.36 (approximately 446 American dollars) [17]. In this city, in 2011, 41.4% of the women giving birth to liveborn infants attended seven or more prenatal visits [18], a lower percentage than that observed for Ribeirão Preto.

### Participants and sample

This was a convenience sample due to the impossibility of obtaining a random sample representative of the population of pregnant women in São Luís and Ribeirão Preto. Pregnant women users of prenatal outpatient clinics of public and private hospital maternities were registered for interview to be held from the 22nd to the 25th week of gestational age. Inclusion criteria were to have performed the first ultrasound exam at less than 20 weeks of gestational age and to intend to give birth at one of the maternities in the municipality where the prenatal interview was held. Multiple pregnancies were excluded.

From February 2010 to June 2011, in São Luís, 1,447 pregnant women participated in the study at the Clinical Research Center (CEPEC in the Portuguese acronym) of the Federal University of Maranhão (UFMA in the Portuguese acronym). One woman was excluded because she did not fill the self-applied questionnaire, leaving 1,446 cases for analysis. In Ribeirão Preto, the sample consisted of 1,400 pregnant women whose data had been collected from February 27, 2010 to February 12, 2011. Data for 1,378 women were used since 22 women did not have complete information about violence.

### Data collection and storage

Two questionnaires were used for data collection: a) the *Self-Applied Prenatal Questionnaire*, to be read and answered by the pregnant women, and b) the *Prenatal Interview Questionnaire*, applied by interviewers. The 13 questions of the WHO VAW for the screening of violence against pregnant women were included in the self-applied questionnaire.

If the pregnant women had doubts about filling out the questionnaire or had reading and writing difficulties, field supervisors helped them. Supervisors and coders reviewed the responses of the interviewees before being typed. Whenever possible, inconsistencies were corrected.

### Instrument for the screening of violence

The questions for the screening of violence during pregnancy were obtained from the Brazilian version of the WHO VAW instrument [19].

For psychological (emotional) violence, women were asked: since you became pregnant has someone V1) insulted you or made you feel bad about yourself?; V2) belittled or humiliated you in front of others?; V3) intimidated or scarred you on purpose?; V4) threatened to hurt you or somebody you care about? [19].

Regarding physical violence during the actual pregnancy women responded to the following questions: since you became pregnant has someone V5) slapped you or thrown something at you that could hurt you?; V6) pushed or shoved you, hit you with a fist or something else that could hurt?; V7) hit you with his/her fist or with some other object that could have hurt you; V8) kicked, dragged or beaten you up?; V9) choked or burnt you on purpose?; V10) threatened you with, or actually used a gun, knife or other weapon against you? [19].

The last three questions dealt with sexual violence: since you became pregnant V11) has someone ever physically forced you to have sexual intercourse against your will?; V12) have you ever had sexual intercourse because you were afraid of what your partner might do?; V13) has someone ever forced you to do something sexual you found degrading or humiliating? [19].

The response options for each of these questions were the following: a) never (coded as zero), b) once (coded as 1), c) a few times (coded as 2), and d) many times (coded as 3) [19].

Raw data from the São Luís and Ribeirão Preto samples are available as S1 and S2 Files in excel format.

### Statistical analysis

Based on the Brazilian version of the WHO VAW instrument [19], 13 observed variables were used (V1 to V13). Latent dimensions psychological violence (considering the four questions about emotional abuse), physical violence (considering the six questions about physical abuse) and sexual violence (considering the three questions about sexual abuse) were hypothesized. The unidimensional model of violence consisted of the 13 observed variables and the multidimensional model consisted of three latent dimensions: emotional, physical and sexual. Cronbach's alpha was calculated in Stata 13.0. Confirmatory factor analysis was performed using the Mplus software, version 7. Since all variables were categorical, the mean-and-variance-adjusted weighted least squares estimator was used.

To determine whether the models showed good fit we considered: a) a p-value (*p*) larger than 0.05 for the Chi-squared test (*χ^2^*) [13]; b) a p-value of less than 0.05 and an upper limit of the 90% confidence interval of less than 0.08 for the Root Mean Square Error of Approximation (RMSEA) [14]; c) values higher than 0.95 for the Comparative Fit Index and the Tucker Lewis Index (CFI/TLI) [14]; and d) Weighted Root Mean Square Residual (WRMR) values of less than 1 [14].

The unidimensional model (Model 1) included the 13 observed variables (V1 to V13) forming the violence construct.

The multidimensional (Model 2) followed the WHO VAW proposal. At the first level, the latent dimensions psychological violence, physical violence and sexual violence were analyzed based on their observed variables. At the second level, it was determined whether these three latent dimensions formed the violence construct. From this step onward, the **modindices** command was used for suggestions of modifications of the initial hypothesis. When the proposed modifications were considered to be plausible from a theoretical viewpoint, a new model was elaborated and analyzed. The **difftest** was used to calculate the difference between the chi-squared values of the models [14].

Models were fitted separately for each city and a third model combining data from the two settings was also tested.

### Ethics Statement

The present investigation was approved by the Research Ethics Committees of the University Hospital of the Federal University of Maranhão (protocol n° 4771/2008-30) and of the University Hospital of the Faculty of Medicine of Ribeirão Preto (protocol n° 4116/2008). The investigators declare that they have no conflicts of interest.

All women gave written informed consent to participate in the study and for those younger than 18 an accompanying adult also signed the consent form. All subjects were informed that the BRISA prenatal cohort was investigating risk factors for preterm birth, and that confidentiality, image protection and non-stigmatization were guaranteed to all participants.

## Results

Cronbach's alpha was 0.76 for general violence, 0.76 for psychological, 0.76 for physical and 0.65 for sexual violence in São Luís. For Ribeirão Preto, it was 0.82 for general violence, 0.79 for psychological, 0.85 for physical and 0.82 for sexual violence. For the combined model including both cities, it was 0.80 for general violence, 0.78 for psychological, 0.82 for physical and 0.78 for sexual violence.

**Figure 1:**
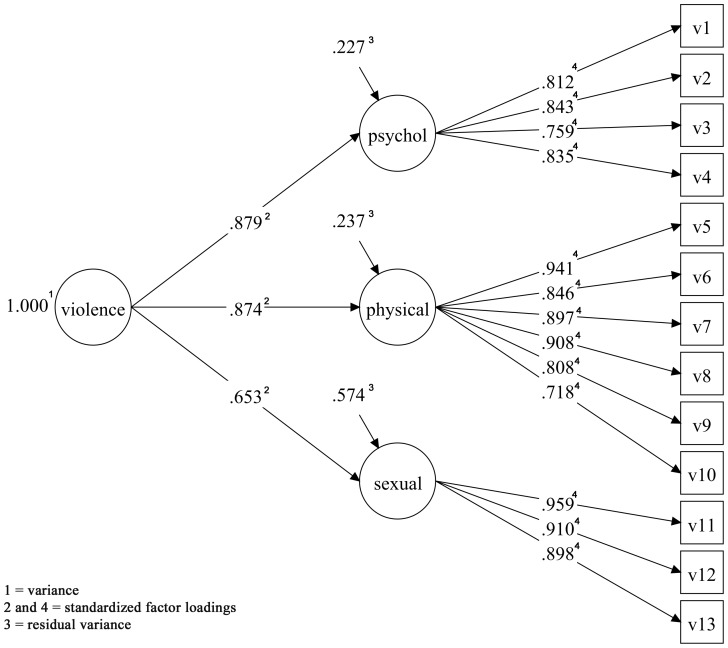
Confirmatory factor analysis of the multidimensional WHO VAW model for pregnant women of the BRISA prenatal cohort of São Luís, Brazil, 2010–2011.

In São Luís, the unidimensional model (Model 1) did not fit the data well by any of the indices adopted (RMSEA = 0.061, CFI = 0.933, TLI = 0.920, WRMR = 1.984). The multidimensional model following the WHO VAW (Model 2) showed good fit (RMSEA = 0.028, CFI = 0.986, TLI = 0.982, WRMR = 0.962) (Table 1). The standardized estimates of the factor loadings included in the three latent dimensions psychological, physical and sexual violence were all higher than 0.7 and statistically significant (all *p*<0.001), and when these three dimensions formed the violence construct, the sexual violence construct presented a factor loading a little below 0.70 (0.65) (Table 2 and Fig. 1).

**Table 1:** Fit indices for the unidimensional and multidimensional models, São Luís-Brazil, 2010/2011.

Indices	Model 1^a^	Model 2^b^	Model 3^c^	Model 4^d^
*χ^2^* ^e^	410.796	134.413	110.586	93.519
Degrees of freedom	65	62	61	60
*p*	<0.001	<0.001	0.001	0.003
RMSEA^f^	0.061	0.028	0.024	0.020
90% CI^g^	0.055–0.066	0.022–0.035	0.016–0.031	0.011–0.027
*p*	0.001	0.999	0.999	0.999
CFI^h^	0.933	0.986	0.990	0.994
TLI^i^	0.920	0.982	0.988	0.992
WRMR^j^	1.984	0.962	0.848	0.756

a Unidimensional model with the 13 variables.

b Model following the WHO Multi-country Study.

c V4 loading in the latent psychological and physical violence factors.

d V3 loading in the latent psychological and physical violence factors.

e Chi-squared test.

f Root Mean Square Error of Approximation.

g Confidence Interval.

h Comparative Fit Index.

I Tucker Lewis Index.

j Weighted Root Mean Square Residual.

**Table 2:** Factor loadings and coefficients of determination for the multidimensional models 2, 3 and 4, São Luís-Brazil, 2010/2011.

Dimensions	Model 2^b^	Model 3^c^	Model 4^d^
	Standardized factor loadings; p value	Standardized factor loadings; p value	Standardized factor loadings; p value
Psychological			
V1	0.812; <0.001	0.824; <0.001	0.843; <0.001
V2	0.843; <0.001	0.858; <0.001	0.881; <0.001
V3	0.759; <0.001	0.778; <0.001	0.533; <0.001
V4	0.835; <0.001	0.513; <0.001	0.446; <0.001
Physical			
V3	-	-	0.281; <0.001
V4	-	0.356; <0.001	0.443; <0.001
V5	0.941; <0.001	0.940; <0.001	0.939; <0.001
V6	0.846; <0.001	0.848; <0.001	0.847; <0.001
V7	0.897; <0.001	0.897; <0.001	0.895; <0.001
V8	0.908; <0.001	0.909; <0.001	0.908; <0.001
V9	0.808; <0.001	0.810; <0.001	0.811; <0.001
V10	0.718; <0.001	0.720; <0.001	0.718; <0.001
Sexual			
V11	0.959; <0.001	0.959; <0.001	0.959; <0.001
V12	0.910; <0.001	0.909; <0.001	0.909; <0.001
V13	0.898; <0.001	0.898; <0.001	0.898; <0.001
Violence Construct			
Psychological Dimension	0.879; <0.001	0.842; <0.001	0.792; <0.001
Physical Dimension	0.874; <0.001	0.844; <0.001	0.819; <0.001
Sexual Dimension	0.653; <0.001	0.670; <0.001	0.690; <0.001
	r^2e^	r^2^	r^2^
Violence Construct			
Psychological Dimension	0.773	0.709	0.628
Physical Dimension	0.763	0.713	0.671
Sexual Dimension	0.426	0.449	0.476

b WHO Multi-country Study Model.

c V4 loading in the latent psychological and physical violence factors.

d V3 loading in the latent psychological and physical violence factors.

e Coefficient of determination.

The highest suggested modification index (25.502) for the WHO VAW model was to include V4 in the physical violence dimension. This modification was considered to be theoretically plausible, forming the multidimensional model 3 (Model 3). This modification resulted in a significant improvement compared to the WHO VAW Model when considering the difference between chi-squares, p-value<0.001 (Table 1). In this model, with V4 being simultaneously part of the psychological and physical violence dimensions, the V4 factor loading was 0.51 for the psychological dimension and 0.35 for the physical dimension (Table 2).

The highest suggested modification index (16.645) suggested for Model 3 was to include V3 in the physical violence dimension. We tested the former modification for being considered plausible (Model 4). The fit of this model was also superior to the WHO VAW Model (*p*<0.001) (Table 1). As a modification to this model, V5 was suggested to load also in the psychological violence, a path that was not considered theoretically plausible.

**Figure 2:**
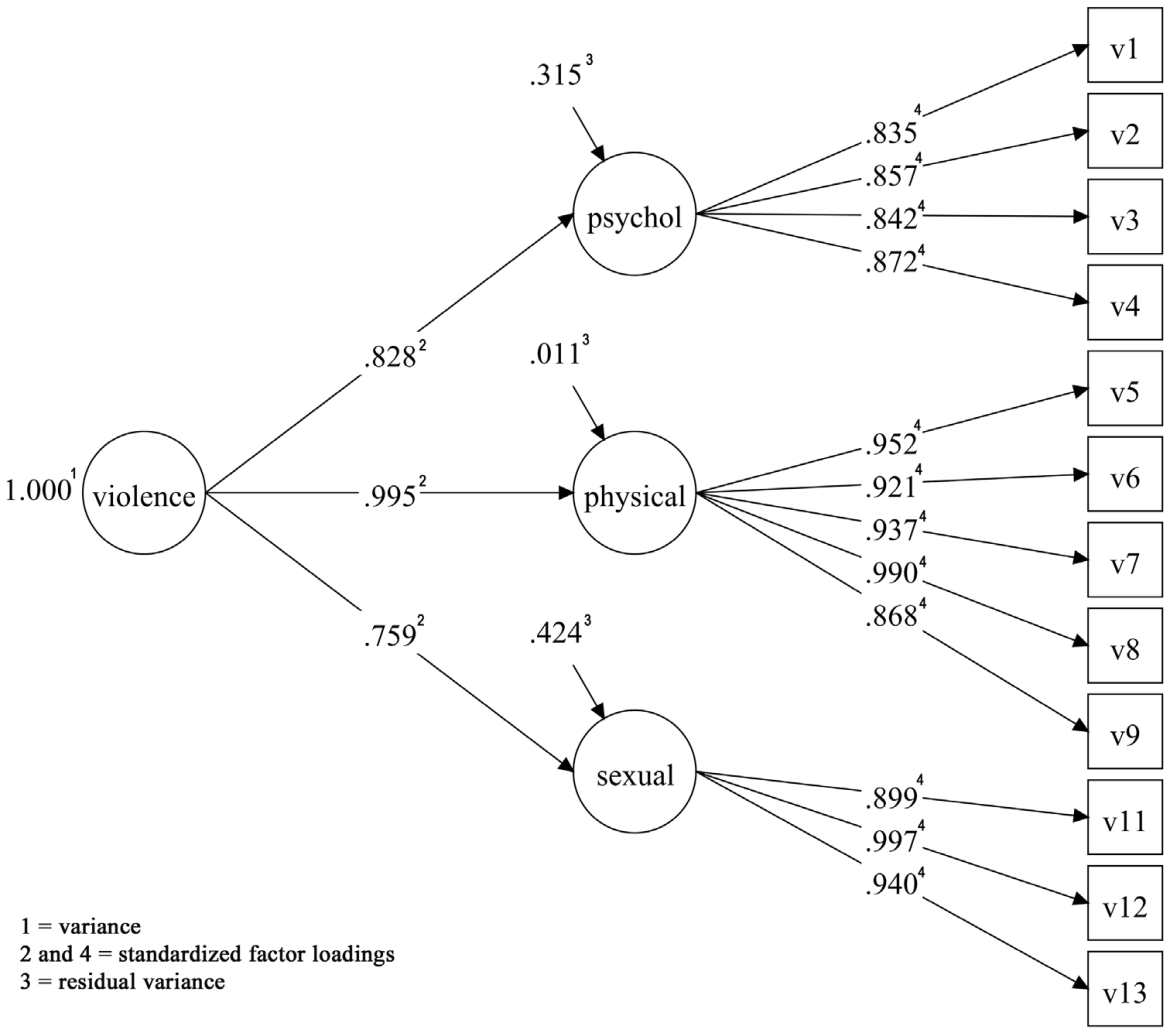
Confirmatory factor analysis of the multidimensional WHO VAW model for pregnant women of the BRISA prenatal cohort of Ribeirão Preto, Brazil 2010–2011.

In Ribeirão Preto, the unidimensional model also did not show good fit (RMSEA = 0.052, WRMR = 1.807). The multidimensional WHO VAW was tested with the Ribeirão Preto data, and showed a good fit (RMSEA = 0.035, CFI = 0.989, TLI = 0.987, WRMR = 0.992) (Table 3 and Fig. 2). The highest modifications indexes suggested for models 2 and 3 in Ribeirão Preto were the same as those suggested for São Luís. The modification index suggested for model 4 in Ribeirão Preto was a crossloading of V6 on sexual dimension, what was considered implausible based on theory. Models 3 and 4, suggested by the modification indices, showed significantly superior adjustment compared to the original WHO VAW model, all p values <0.001 (Table 4).

**Table 3:** Fit indices for the unidimensional and multidimensional models, Ribeirão Preto-Brazil, 2010/2011.

Indices	Model 1^a^	Model 2^b^	Model 3^c^	Model 4^d^	Model 2 without V10
*χ^2^* ^e^	311.365	163.863	117.766	100.497	132.569
Degrees of freedom	65	62	61	60	51
*p*	<0.001	<0.001	<0.001	0.008	<0.001
RMSEA^f^	0.052	0.035	0.026	0.022	0.034
90% CI^g^	0.047–0.058	0.028–0.041	0.019–0.033	0.014–0.030	0.027–0.041
*P*	0.237	0.999	0.999	0.999	0.999
CFI^h^	0.974	0.989	0.994	0.996	0.991
TLI^i^	0.969	0.987	0.992	0.994	0.989
WRMR^j^	1.807	0.992	0.807	0.728	0.907

a Unidimensional model with the 13 variables.

b Model following the WHO Multi-country Study.

c V4 loading in the latent psychological and physical violence factors.

d V3 loading in the latent psychological and physical violence factors.

e Chi-squared test.

f Root Mean Square Error of Approximation.

g Confidence Interval.

h Comparative Fit Index.

I Tucker Lewis Index.

j Weighted Root Mean Square Residual.

**Table 4:** Factor loadings and coefficients of determination for the multidimensional models 2, 3 and 4, Ribeirão Preto-Brazil, 2010/2011.

Dimensions	Model 2^b^	Model 3^c^	Model 4^d^	Model 2 without V10
	Standardized factor loadings; p value	Standardized factor loadings; p value	Standardized factor loadings; p value	Standardized factor loadings; p value
Psychological Dimension				
V1	0.831; <0.001	0.847; <0.001	0.867; <0.001	0.835;<0.001
V2	0.855; <0.001	0.871; <0.001	0.892; <0.001	0.857;<0.001
V3	0.842; <0.001	0.869; <0.001	0.612; <0.001	0.842;<0.001
V4	0.883; <0.001	0.401; <0.001	0.348; <0.001	0.872;<0.001
Physical Dimension				
V3	-	-	0.274; <0.001	-
V4	-	0.487; <0.001	0.550; <0.001	-
V5	0.948; <0.001	0.948; <0.001	0.947; <0.001	0.952;<0.001
V6	0.917; <0.001	0.918; <0.001	0.918; <0.001	0.921;<0.001
V7	0.935; <0.001	0.935; <0.001	0.935; <0.001	0.937;<0.001
V8	0.986; <0.001	0.986; <0.001	0.985; <0.001	0.990;<0.001
V9	0.877; <0.001	0.877; <0.001	0.877; <0.001	0.868;<0.001
V10	0.898; <0.001	0.897; <0.001	0.898; <0.001	-
Sexual Dimension				
V11	0.906; <0.001	0.906; <0.001	0.906; <0.001	0.899;<0.001
V12	0.992; <0.001	0.991; <0.001	0.991; <0.001	0.997;<0.001
V13	0.941; <0.001	0.941; <0.001	0.941; <0.001	0.940;<0.001
Violence Construct				
Psychological Dimension	0.816; <0.001	0.758; <0.001	0.714; <0.001	0.828;<0.001
Physical Dimension	1.018; <0.001	1.025; <0.001	1.020; <0.001	0.995;<0.001
Sexual Dimension	0.769; <0.001	0.764; <0.001	0.765; <0.001	0.759;<0.001
	r^2e^	r^2^	r^2^	r^2^
Violence Construct				
Psychological Dimension	0.666	0.574	0.509	0.685
Physical Dimension	undefined	undefined	undefined	0.989
Sexual Dimension	0.591	0.584	0.585	0.576

b Model following the WHO Multi-country Study.

c V4 loading in the latent psychological and physical violence factors.

d V3 loading in the latent psychological and physical violence factors.

e Coefficient of determination.

Analysis of the Ribeirão Preto data revealed a negative value for the residual variance of the physical violence dimension, generating an improper solution (Heywood case). This Heywood case was probably provoked by data idiosyncrasy. For this reason, the variable V10 was excluded from the original WHO-VAW to test for consistency. The exclusion of V10 produced adequate estimates results were similar compared to models including V10 (Table 4, last column).

**Figure 3:**
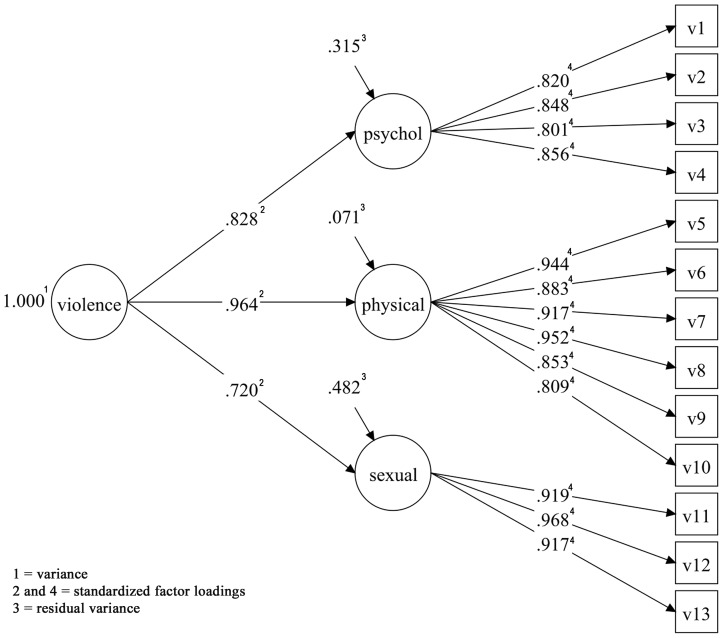
Confirmatory factor analysis of the multidimensional WHO VAW model for pregnant women of the BRISA prenatal cohort, combined data from São Luís and Ribeirão Preto, Brazil 2010-2011.

For the combined model, the unidimensional model also did not show good fit (RMSEA = 0.060, WRMR = 2.570). The multidimensional WHO VAW was tested and showed a good fit (RMSEA = 0.036, CFI = 0.983, TLI = 0.979, WRMR = 1.312), although the WRMR was found to be a little above the suggested cut-off point (Table 5 and Fig. 3). The highest modifications indexes suggested for models 2 and 3 were the same as those suggested for São Luís and Ribeirão Preto. Models 3 and 4, suggested by the modification indices, showed significantly superior adjustment compared to the original WHO VAW model, all p values <0.001 (Table 6).

**Table 5:** Fit indices for the combined model, São Luís and Ribeirão Preto-Brazil, 2010/2011.

Indices	Model 1^a^	Model 2^b^	Model 3^c^	Model 4^d^
*χ^2^* ^e^	729.757	283.349	211.428	163.813
Degrees of freedom	65	62	61	60
*p*	<0.001	<0.001	<0.001	<0.001
RMSEA^f^	0.060	0.036	0.030	0.025
90% CI^g^	0.056–0.064	0.031–0.040	0.025–0.034	0.021–0.030
*p*	0.999	0.999	0.999	0.999
CFI^h^	0.949	0.983	0.988	0.992
TLI^i^	0.939	0.979	0.985	0.989
WRMR^j^	2.570	1.312	1.110	0.974

a Unidimensional model with the 13 variables.

b Model following the WHO Multi-country Study.

c V4 loading in the latent psychological and physical violence factors.

d V3 loading in the latent psychological and physical violence factors.

e Chi-squared test.

f Root Mean Square Error of Approximation.

g Confidence Interval.

h Comparative Fit Index.

I Tucker Lewis Index.

j Weighted Root Mean Square Residual.

**Table 6:** Factor loadings and coefficients of determination for combined model, São Luís and Ribeirão Preto-Brazil, 2010/2011.

Dimensions	Model 2^b^	Model 3^c^	Model 4^d^
	Standardized factor loadings; p value	Standardized factor loadings; p value	Standardized factor loadings; p value
Psychological			
V1	0.820; <0.001	0.834; <0.001	0.855; <0.001
V2	0.848; <0.001	0.863; <0.001	0.886; <0.001
V3	0.801; <0.001	0.823; <0.001	0.565; <0.001
V4	0.856; <0.001	0.472; <0.001	0.409; <0.001
Physical			
V3	-	-	0.285; <0.001
V4	-	0.404; <0.001	0.483; <0.001
V5	0.944; <0.001	0.943; <0.001	0.942; <0.001
V6	0.883; <0.001	0.885; <0.001	0.884; <0.001
V7	0.917; <0.001	0.917; <0.001	0.917; <0.001
V8	0.952; <0.001	0.952; <0.001	0.951; <0.001
V9	0.853; <0.001	0.853; <0.001	0.853; <0.001
V10	0.809; <0.001	0.810; <0.001	0.811; <0.001
Sexual			
V11	0.919; <0.001	0.919; <0.001	0.919; <0.001
V12	0.968; <0.001	0.968; <0.001	0.968; <0.001
V13	0.917; <0.001	0.917; <0.001	0.917; <0.001
Violence Construct			
Psychological Dimension	0.828; <0.001	0.782; <0.001	0.735; <0.001
Physical Dimension	0.964; <0.001	0.950; <0.001	0.933; <0.001
Sexual Dimension	0.720; <0.001	0.730; <0.001	0.740; <0.001
	r^2e^	r^2^	r^2^
Violence Construct			
Psychological Dimension	0.685	0.612	0.541
Physical Dimension	0.929	0.902	0.870
Sexual Dimension	0.518	0.532	0.547

b WHO Multi-country Study Model.

c V4 loading in the latent psychological and physical violence factors.

d V3 loading in the latent psychological and physical violence factors.

e Coefficient of determination.

## Discussion

The present study, by applying and validating the WHO VAW instrument in two Brazilian cities with confirmatory factor analysis, underscores the need to consider violence against pregnant women as a multidimensional phenomenon, with its psychological, physical and sexual sub-scales.

The unidimensional models (combined, including both cities and separated models for each city) did not present good fit, showing that the 13 variables of the WHO VAW instrument did not form a single violence scale. These findings support the view that violence against pregnant women is a complex multidimensional phenomenon [2], [3].

The multidimensional second-order models (combined, including both cities and separated models for each city) as proposed by the WHO VAW showed good adjustment, confirming what had already been shown in studies that used exploratory factor analysis [11], [12].

The WHO VAW model was improved by the modification indices proposed, indicating that questions V3 (has someone ever intimidated or scarred you on purpose) and V4 (has someone ever threatened to hurt you or somebody you care about) seem to measure simultaneously the psychological and physical dimensions of violence. This was noted for the combined model including the two cities and also for the two separated models fitted for each city. Overlap of questions regarding psychological and physical violence had already been suggested by the first validation study of the WHO VAW questionnaire, with questions V5 and V6 (pushed or shoved you, hit you with a fist or something else that could hurt) of physical violence showing cross loadings with psychological violence only in one of the two sites studied, i.e., the Wooded Zone of Pernambuco [11].

Cross loadings of sexual violence and the other dimensions of psychological and physical violence were also observed in the Brazilian [11], but not in the present study. Differences in study samples may have possibly contributed to these findings since we only interviewed pregnant women, while the WHO Multi-country Study also included non-pregnant women.

It is important to note that Cronbach's alpha for sexual violence was higher for the Ribeirão Preto sample than for the São Luís sample. We hypothesize that the way in which sexual abuse questions were asked in the WHO VAW questionnaire was closer to its cultural meaning or to the actual experience of this form of abuse in the Ribeirão Preto sample.

The use of a convenience sample limits the external validity of the findings. It is unlikely that recall bias occurred in the responses to violence questions during the gestational period, since this period is short and the data were collected during the second trimester of pregnancy.

A differential aspect of the present study was the validation of the WHO VAW instrument according to the multidimensional theory of violence for use in the self-applied form by pregnant women from different socioeconomic contexts, by means of confirmatory factor analysis.

Thus, violence is a multidimensional second-order construct consisting of psychological, physical and sexual dimensions. The WHO VAW models and the modified ones showed good fit and are suitable to measure violence against pregnant women. Considering that the model proposed by the WHO is more parcimonious and also showed good fit, it could be preferentially used in the measurement of violence against women.

The use of a validated questionnaire containing questions about psychological, physical and sexual violence can help the professionals who provide prenatal care to better screen for this phenomenon.

## Supporting Information

S1 File Raw data for the São Luís sample.
(XLSX)

S2 File Raw data for the Ribeirão Preto sample.
(XLSX)
